# A human-centered approach for sharing patient experiences through digital storytelling: a research through design study

**DOI:** 10.1017/dsj.2024.26

**Published:** 2024-10-25

**Authors:** Sana Behnam-asl, Kelly Umstead, Raunak Mahtani, Kristin P. Tully, Carolina Gill

**Affiliations:** 1 College of Design, North Carolina State University, Raleigh, NC, USA; 2 Department of Obstetrics and Gynecology, School of Medicine, University of North Carolina at Chapel Hill, Chapel Hill, NC, USA

**Keywords:** Health care, Human-centered approach, Design research, Research through design, Digital storytelling

## Abstract

This article outlines a human-centered approach to developing digital patient stories, for sharing their experiences in health care, while preserving patient and others’ privacy. Employing a research-through-design approach, the study proposes a design solution using visualization and digital storytelling to document patients’ and families’ experiences and emotions, as well as their interactions with healthcare professionals in the postnatal unit. By transforming selected observational data into animated stories, this approach has the potential to elicit empathy, stimulate stakeholder engagement, and serve as a practical training tool for clinicians. This work was conducted as part of a broader study that aims to contribute to the existing knowledge base by advancing our understanding of stakeholder needs in birthing facilities and through postpartum discharge. This study primarily focuses on strategies for the development of digital stories and summarizes the factors that contributed to the production of digital stories within the context of sensitive data. It may serve as a valuable resource for students, researchers and practitioners interested in utilizing digital stories to encourage discussions, education and ultimately to enhance systems of health care for respect, equity and support.

## 1. Introduction

Designers are playing an increasingly active role in the healthcare sector, leveraging design-based research, visualization and prototyping methods to reimagine systems of care (Partridge 2017; Tsekleves & Cooper 2017; Mahtani et al. 2022; Lamé et al. 2023). This trend has been accompanied by the recognition and adoption of human-centered design (HCD) within the healthcare industry (Bazzano et al. 2017; Göttgens & Oertelt-Prigione 2021; Melles, Albayrak, & Goossens 2021), which places the human at the center of the research and development process of interventions, using techniques such as visualization, communication between researchers and stakeholders, empathy and simulation to better understand and address the needs of all stakeholders (Bazzano et al. 2017; Bjerkan et al., 2015; Giacomin 2014; Gill et al. 2023; Hridi et al. 2022; Saedi & Rice 2020; Saedi & Rice 2021).

HCD is a systematic approach to innovative problem solving that requires meaningful engagement of stakeholders, collaboration, team effort and the integration of multidisciplinary skills and perspectives for refining solutions and designs through iterative feedback and user testing (Harte et al. 2014, 2017; Li et al. 2018; ISO 2019).

In health care, a human-centered perspective is crucial for understanding the unique needs, behaviors, expectations and lived experiences of target users and key stakeholders when assessing clinical strengths and gaps and co-developing health interventions (Barello et al. 2016; Fleisher et al. 2020). Maternal and postnatal health is a multifaceted and complex area (Arzamani et al. 2022), where HCD can be particularly effective by addressing existing disparities and promoting equitable access to care. The United States faces significant maternal and postnatal health problems due to factors such as racial biases, fragmented care and limited patient-focused support or education on maternity-related complications and health (CDC 2021; Petersen et al. 2019b; Howell 2018). Prompt recognition of maternal warning signs and risk appropriate responses in structurally competent healthcare systems could prevent two-thirds of maternal deaths (CDC 2019; Petersen et al. 2019a; Howell 2018). By prioritizing human-centered views, health interventions can be developed that are tailored to the unique requirements of the target stakeholders, leading to increased adoption, engagement and ultimately, improved health outcomes (Bazzano et al. 2017; ISO 2019). This partnership is particularly important for addressing the maternal mortality and morbidity crises and improving maternity care-related outcomes (Jaynes, Brathwaite, & Tully 2022).

This study was one part of a multiphase, mixed methods study. The project aimed at generating new knowledge to inform improvements in maternal and postpartum healthcare systems. The investigators included designers, clinicians, researchers and engineers collaborating across three large research universities. The design research team was composed of two senior industrial design faculty members and two industrial design PhD students. The primary goal of this aspect of the research was to generate new knowledge to advance the understanding of stakeholder needs on the postnatal unit. The research partners included physicians and experts from fields such as, pediatrics, medical anthropology, clinical research, health equity, midwifery, nursing, maternal health, research management, health informatics, public health, epistemology, patient education, science communications, clinical implementation and infant health. In this article, we will refer to the design team as “design researchers”, collaborators and partners as “research partners” and the entire team as the “research team”. This work led to the generation of novel ways to conduct research and apply their skills, tools and methods for impact.

The research partners employed a multi-method approach to collect observational data, including partnership with new families and their healthcare team members to document their experiences on a postnatal unit. The video and audio recordings provided direct accounts of the participants’ emotional, behavioral and interactional dynamics during their postnatal stay and hospital discharge, recording moments of supportive and compassionate care as well as challenging situations that arose among patients, their families and healthcare professionals. The data offered unique insights into experiences that would have otherwise been difficult to convey, document and analyze.

Although such research data could potentially be directly used for clinician training and education, this type of data includes many issues that make this application inappropriate (Iedema et al. 2006a, 2006b; Asan & Montague 2014). Naturalistic recordings contain extensive personal identifying information and the length of the videos, even for interactions that take place over minutes of time, would be too long for teaching and training purposes.

The data were initially collected to identify patterns of behavior, context and interactions in the postnatal unit. The richness of this dataset subsequently provided opportunities for design to effectively convey these important findings.

### 1.1. Aims

Using a human-centered perspective and a research-through-design (RtD) approach, this study proposed a design solution that leveraged visualization and digital storytelling to share selected experiences and emotions of patients and families in the postnatal unit while preserving the privacy of all participants (Seshadri et al. 2019). Transforming parts of the recordings into animated stories had the potential to evoke empathy, encourage stakeholder engagement and serve as a practical training tool for clinicians (Sitter, Beausoleil, & McGowan 2020; Lambert & Brooke 2018). The research process utilized in this study was documented to inform frameworks for digital story development in the context of health care. In particular, contributing factors to the production of digital stories within the context of sensitive data were summarized. Certain sections were condensed to create concise digital narratives (typically lasting between 2 and 3 minutes), each focusing on a key issue. The primary emphasis of this article lies in the creation and evolution of the digital stories, rather than their subsequent implementation. This article can serve as a resource for students, researchers and practitioners interested in exploring the development of digital stories as a means of encouraging and provoking discussions on complex health-related topics to enhance care.

### 1.2. Naturalistic videos and digital storytelling

Naturalistic video refers to recording the real-time actions and verbalizations of individuals in their environment through unobtrusive video recording, with the purpose of systematically coding, analyzing and disseminating their experiences (Pink 2007). Naturalistic videos have been used to observe complex social and health-related settings, including maternity care, to address patient safety and clinical efficiency (e.g., Iedema et al. 2006b; Tully & Ball 2012; Asl et al. 2014). The use of video and audio data from clinical encounters can offer insights into the nuanced dynamics of these interactions, as well as the expressed emotions and reactions of patients and their companions. This information can be used to identify areas of improvement in patient care and provide positive examples of interactions between healthcare professionals and patients (e.g., Bensing, Verheul, & Van Dulmen 2008; Tully & Ball 2012). However, the possibility of using these videos as educational material for training healthcare professionals presents specific challenges due to the sensitive and confidential nature of the information. Obtaining consent from participants to share the videos outside of the study team may substantially limit enrollment and affect participation, while also introducing ethical management complexities (Everri et al. 2020). Additionally, traditional formats for sharing qualitative data, such as accounts of interactions and representative quotes, may not fully convey the emotional and contextual information present in the videos (Evans et al. 2019).

Digital storytelling offers an opportunity to appropriately and meaningfully share behaviors, contexts, nonverbal cues and environmental factors from observational data, providing important insight into the structure, content and emotions of clinical encounters. Digital storytelling has emerged as an art-based powerful method of conveying personal experiences in an engaging and compelling way (Lambert & Brooke 2018). It involves the creation of brief narratives, typically lasting between 3 and 5 minutes, using a combination of images, video, audio and text (Gubrium 2009; Stenhouse et al. 2013; Matthews & Sunderland 2017).

This method has been extensively employed in health care, education, advocacy and knowledge transfer (Sitter et al. 2020). Moreover, the approach has undergone significant development, incorporating various lengths, processes and technological formats. These lessons learned inform current tools for fostering engagement, persuasion, participatory visual research and behavior change (Boydell et al. 2012; Scott et al. 2013). Focusing on the healthcare sector, digital storytelling has proven to be an effective tool for sharing culturally relevant and respectful information and messages. It provides an accessible way to engage patients in research and disseminate experiential knowledge to a larger audience (Gray, Young, & Blomfield 2015; Lal, Donnelly, & Shin 2015; Hardy 2016; Moreau et al. 2018). Healthcare practitioners, educators, advocates and researchers have successfully partnered with community members to create powerful digital stories that serve as a participatory visual research method, and their effectiveness has been widely recognized and documented in literature (Cueva et al. 2016; Gubrium et al. 2016; de Jager et al. 2017).

In the context of maternity care, digital stories and narrative-based videos have been found to promote various behavioral changes and enhance well-being, including managing stress and weight, increasing awareness, encouraging physical activity, adopting healthy lifestyles and improving communication practices (Evans et al. 2019; Haith-Cooper et al. 2020; Parsons et al. 2021; Farao 2023). Additionally, studies have shown that digital storytelling can be a powerful tool for enhancing empathy and understanding among healthcare professionals, particularly in areas such as neonatal care and midwifery. For example, Charon (2004) emphasizes the concept of “narrative competence” as a valuable skill for medical practitioners, highlighting the significance of being responsive toward emotional needs of others in medical context.

Moreover, arts-based digital storytelling based on parents’ experiences in neonatal care has been found to enhance empathy in nursing students (Petty 2023). Similarly, Thompson, Lamont-Robinson, & Younie (2010) advocate for the inclusion of artistic creativity within the medical curriculum, suggesting that fostering creativity is essential for developing well-rounded and emotionally intelligent medical practitioners. In addition to enhancing understanding, digital storytelling has the potential to improve communication skills, ethical practice and cultural competence among healthcare professionals. By accessing personal narratives and experiences, healthcare professionals can gain valuable insights into the emotional and contextual aspects of patient care (Petty, Jarvis, & Thomas 2020), which may lead to more compassionate and effective healthcare delivery.

Similarly, Kalra et al. (2022) implemented a quality improvement program in Bihar, India, to enhance intrapartum care in public health facilities. Nurses participated in simulation sessions complemented by a digital interactive comic series featuring a simulation facilitation superhero called “Super Divya,” aimed at reinforcing key concepts and strengthening the simulation educator’s facilitation skills. The study involved 205 simulation educators who received comic modules, with pre-post surveys conducted with each module. The results showed significant improvements in the knowledge of simulation educators regarding concepts important for successfully conducting simulation training.

Digital storytelling has the potential to significantly enhance medical education and healthcare delivery by providing an engaging, empathetic and culturally sensitive approach to learning and practice. By integrating digital storytelling into medical curricula and professional development programs, healthcare professionals can enhance their skills, improve patient outcomes and contribute to a more compassionate and patient-centered healthcare system (Petty et al. 2020; Kalra et al. 2022; Noya, Oguro, & Horiuchi 2022; Petty 2023).

However, despite its potential as an educational and health promotion tool in the context of maternal health care, the use of digital storytelling in this context is underexplored. Therefore, this study aims to illuminate the role of digital storytelling in generating knowledge and provoking discussion around maternal health care, while exploring its potential for promoting collaboration and improving systems of care.

### 1.3. Research approach

This study employed a Research through Design approach (Stappers & Giaccardi 2014), which involved combining the processes of designing and conducting user research using prototypes and iterative engagement of stakeholders. Knowledge was gathered through the creation of design concepts and prototypes and evaluation of these prototypes with the research partners.

#### Research through design

RtD is an approach that employs design and design thinking as key elements in generating new knowledge and addressing research questions (Koskinen et al. 2013). This research approach draws upon principles and practices from various design fields, including product design, architecture, interaction design and graphic design, among others. RtD involves creating and testing design artifacts or interventions in real-world contexts as a means of exploring research problems and generating new insights (Stolterman & Wiberg 2010). The focus of RtD is not only on the final design product, but also on the design process itself and the knowledge gained through it. RtD primarily focuses on exploring and understanding design problems and opportunities through the process of designing and creating artifacts, such as prototypes or concepts. The prototypes and concepts helped to identify questions that were not apparent previously. In RtD, the approach can start with varying steps depending on the specific goals of the research and the preferences of the researchers or designers involved. However, generally speaking, RtD typically begins with a design problem or opportunity rather than with predefined research questions (Zimmerman, Forlizzi, & Evenson 2007). RtD is particularly relevant in contexts where traditional research methods may not be adequate or where new solutions to complex problems are needed (Stappers & Giaccardi 2014). Given the potential of RtD, researchers can utilize this approach for addressing complex problems and finding patterns about different aspects of the phenomenon using appropriate RtD framework (Stolterman & Wiberg 2010). RtD has three practices or frameworks: lab, field (participatory design and user-centered design) and showroom (critical design).

The lab framework is based on conducting research in a specific environment with users and allows designers to ideate, explore and test ideas. The field framework focuses on the context and is conducted with users within the practice environment. Through this intentional approach, researchers can focus on determining what people do in real life rather than what they say they do. The showroom framework follows a critical design approach and involves an iterative process of defining and redefining the problem space and generating forms and artifacts that provoke people’s imaginations and reactions. Through this approach, design or research goes beyond creating solutions for a single problem and initiates new thoughts and perspectives. All of these frameworks share similar core values of design research and RtD (Koskinen et al. 2013; Olson and Kellogg 2014; Zimmerman, Stolterman, & Forlizzi 2010). Hence, the research question can inform the appropriate framework of the RtD to address the specific objectives (Stappers & Giaccardi 2014).

#### Characteristics and assumptions

The concept of “research through design” was introduced by Christopher Frayling in 1993, in which he described three ways research can be related to design: “research into design”, “research for design” and “research through design” (Frayling 1993). To better understand the distinction between these terms, this article provides an overview of their goals and boundaries. “Research into design” focuses on studying the human activity of design, such as documenting and studying the objects, phenomena and history of design (Olson & Kellogg 2014; Stappers & Giaccardi 2014). “Research for design” is the process of conducting research as part of design practice, gathering and applying relevant scientific and technological information to guide and develop design practice (Stappers & Giaccardi 2014). “Research through design” is a research approach focused on creating new things to improve the world, relying on an empathic understanding of stakeholders, synthesis of behavioral theory and current and near-current technology to envision a desired future (Zimmerman et al. 2010).

In the context of research, RtD is based on the characteristics of design and its reflective and cyclical processes. It acknowledges the potential of designerly ways of knowing and tacit knowledge in addressing complex research questions and wicked problems related to social justice, sustainability and ethics. Through a rigorous process of defining and redefining the research question, RtD aims to generate new knowledge and create a shared understanding of an imagined future. The RtD process differs from a typical design process in its emphasis on generating new knowledge and insights through the act of designing itself. Unlike a traditional design process, which may prioritize the development of a specific product or solution, RtD is more exploratory and iterative in nature, with a focus on understanding the underlying design problems and opportunities. This differs from other approaches like usability testing, where the questions are already known, and researchers use the design process to seek answers. Making and critiquing artifacts is an essential aspect of the RtD process, drawing on principles of design and designerly ways of knowing (Cross 1982; Olson & Kellogg 2014). Furthermore, robust documentation of the processes and explorations with artifacts are other significant characteristics of this approach that can help researchers move beyond designing products and broaden their perspectives about new strategies to shape the desired future and develop knowledge (Zimmerman et al. 2007; Bardzell et al. 2016). For instance, Zimmerman & Forlizzi (2014) shared their research endeavors that focused on developing new methods of interaction with objects by combining various aspects of experimental psychology and design practice. They also developed a new approach to the design of work automation that advocated for democracy and worker protection. In this way, RtD can initiate reflective discussions about altering the current reality toward the desired reality.

Zimmerman & Forlizzi (2014) propose a five-step approach to conducting an RtD research project. The first step is to “Select,” which involves choosing a research problem or a design opportunity, selecting a target population, context and material to work with. The selection process is iterative and requires trying different options until the team reaches consensus. Important considerations in selecting a problem space include whether it is a complex issue that can benefit from design thinking, if it has multiple agendas from various stakeholders, and if the research problem is suitable for investigation using RtD. The team should also consider their skills and the desires and concerns of stakeholders when selecting a problem space. The five steps in this approach include “Select,” “Design,” “Evaluate,” “Reflect and Disseminate,” and “Repeat.” Each step is defined in Table 1.

#### RtD in the context of healthcare systems

The complexity of healthcare problems is a prime example of the value of applying a reflective and iterative process of evaluation and problem reframing, which are critical characteristics of RtD. RtD researchers can use their tacit knowledge to define and reinterpret complex problems through empathic understanding of stakeholders, leading to addressing problems related to cultural competence, health inequities and other aspects of quality care. In addition, RtD offers opportunities to ideate and develop human-centered technologies that can improve the quality of life and experience of stakeholders. Prototyping and iterative development processes can actively engage stakeholders in the knowledge creation process and pave the way for finding patterns in complex problem spaces. Through RtD, researchers can bridge the gap between healthcare practices and emerging technologies and develop interventions that can enable stakeholders to achieve their preferred future.

## 2. Methods

### 2.1. Study design

To develop digital stories to encourage discourse among maternity healthcare professionals, this study used an RtD approach. This process includes a combination of field and showroom practices, consisting of the following four phases: selection, design, evaluation and reflection. The reflection phase is detailed in the discussion section of this article.

#### RtD phase 1: selection

In the selection phase, the design researchers aimed to focus the subject matter and determine a medium for design implementation. Improving the quality of postnatal care is recognized as a multifaceted problem owing to the structural complexity and presence of multiple agendas, driven by distinct stakeholders, such as patients, their families and various healthcare professionals. Given the intricacies of maternal health issues and the known inequities in this context, including health disparities, racial biases, fragmented care and limited support, pursuing maternal and postnatal health through HCD is an important and relevant topic.

Prior to this RtD study, the research partners conducted multi-method data collection after review by the UNC Chapel Hill Biomedical Institutional Review Board (#19–1900). The observational filming of this study was a partnership with 15 postpartum patients, their companions and their healthcare team members at an academic medical center in the southeastern United States. Over 460 hours of video and audio data were collected on the postnatal unit from two cameras placed in the participants’ hospital rooms, for an average filming duration of 31 hours and a range of 10–76 hours. The research team used a behavioral taxonomy to code specific behaviors and content, and they created detailed vignettes to provide time-based accounts of events, participants, situations and structures in alignment with Cueva et al. (2015). These vignettes included memos recorded by the research team to identify and reflect on important points in the study of behaviors, perceptions and expressions of the research participants.

The content of these videos and the experiences of the patients and their companions were so compelling, that the design researchers and research partners recognized an opportunity to expand results beyond the quantification of behaviors and qualitative accounts. Therefore, it was decided to use a combination of field and showroom RtD practices to create digital stories based on the data to share selected experiences of postpartum patients and their companions. These digital stories depicting actual events were intended to promote discussions among healthcare team members and professionals, with the ultimate aim of enhancing systems of care.

#### RtD phase 2: design

Through collaborative prioritization of observed scenarios, we developed digital stories which conveyed the emotional atmosphere and empathic aspects of selected postnatal experiences. In total, the research team created 12 digital stories between January 2021 and March 2023. The initial efforts regarding this process have been previously described (Asl et al. 2022), and subsequently, more digital stories were developed as the project progressed.

#### Design of digital stories..

The research partners and design researchers sought to establish a collaborative and multidisciplinary approach to create digital stories that accurately portrayed the observed events. To achieve this, clinicians and other research partners reviewed observational files and vignettes to identify priority interactions, which were then used to create iterations of digital stories to stimulate discourse among healthcare professionals. The design researchers followed a process that included establishing the digital story scope, designing the style and technical considerations.

#### Establishing the digital story scope..

To select the salient interactions and experiences, the design researchers collaborated with research partners to identify appropriate examples and priority areas. A key consideration in establishing the scope was focused on exploring how storytelling and digital stories could facilitate conversations among healthcare professionals for reflection of system strengths and opportunities for improvement. Another key consideration centered on identifying specific dimensions of the patient interactions, experiences and settings that could draw attention to particular issues. To structure this process, the design researchers created a worksheet that allowed the research partners to choose and summarize sections of the video and note examples worth pursuing as digital stories (Table 2). Using the worksheets and referring to the videos and vignettes, the design researchers and research partners defined a specific objective for each potential digital story.

**Figure 1.**
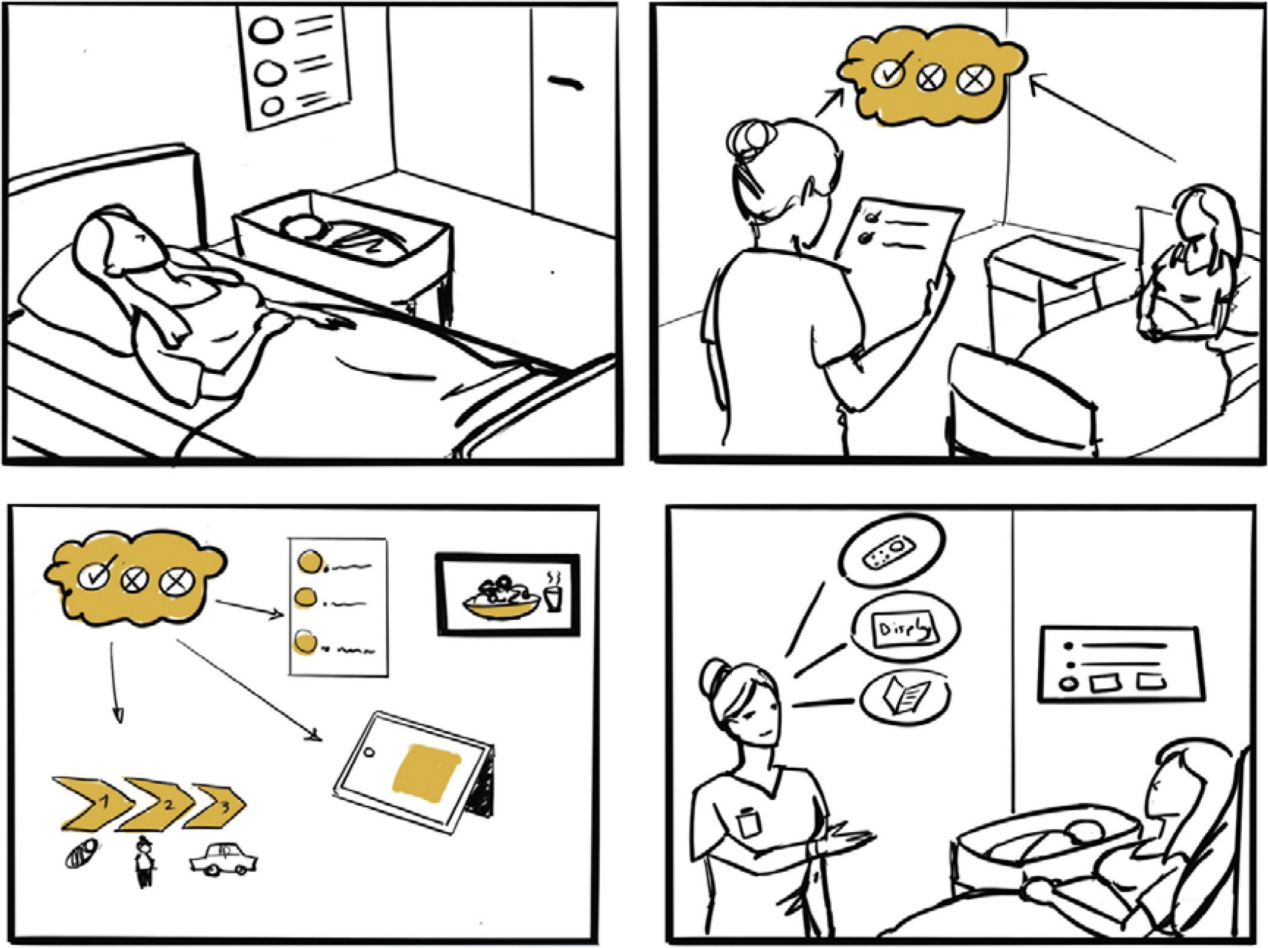
Storyboarding the key ideas of the script to identify essential assets and characters for the digital story.

The design researchers used the information collected from the digital story development worksheets to develop scripts for the animations. The scripts were written to ensure that digital stories were no more than 2–3 minutes, facilitating targeted and effective conversations for education. They focused on key points and events while eliminating any distractions. This process was iterative and conducted in partnership with the research partners due to the complexity of framing the context and finding the appropriate balance regarding details to include in the digital stories. This also allowed for reducing bias and ensuring the credibility and accuracy of the information. Research partners streamlined the stories while maintaining the emotion of the experience. For example, not every part of conversations from the selected scenarios was included as it could have been too long or confusing to the viewers out of context. Additionally, storyboarding based on the key ideas of the script was used throughout the process to determine the essential assets and characters for the story (Figure 1).

The research team also aimed to reduce tangential content to enable the viewers to focus on a certain topic, with preservation of verbatim wording to the extent possible. Additionally, a narrator was included to present an overview of the setting and context at the beginning of each story. The narrator also concluded each video by presenting questions for reflection that invite the audience to engage around the experiences depicted in the digital stories.

#### Visual design..

**Figure 2.**
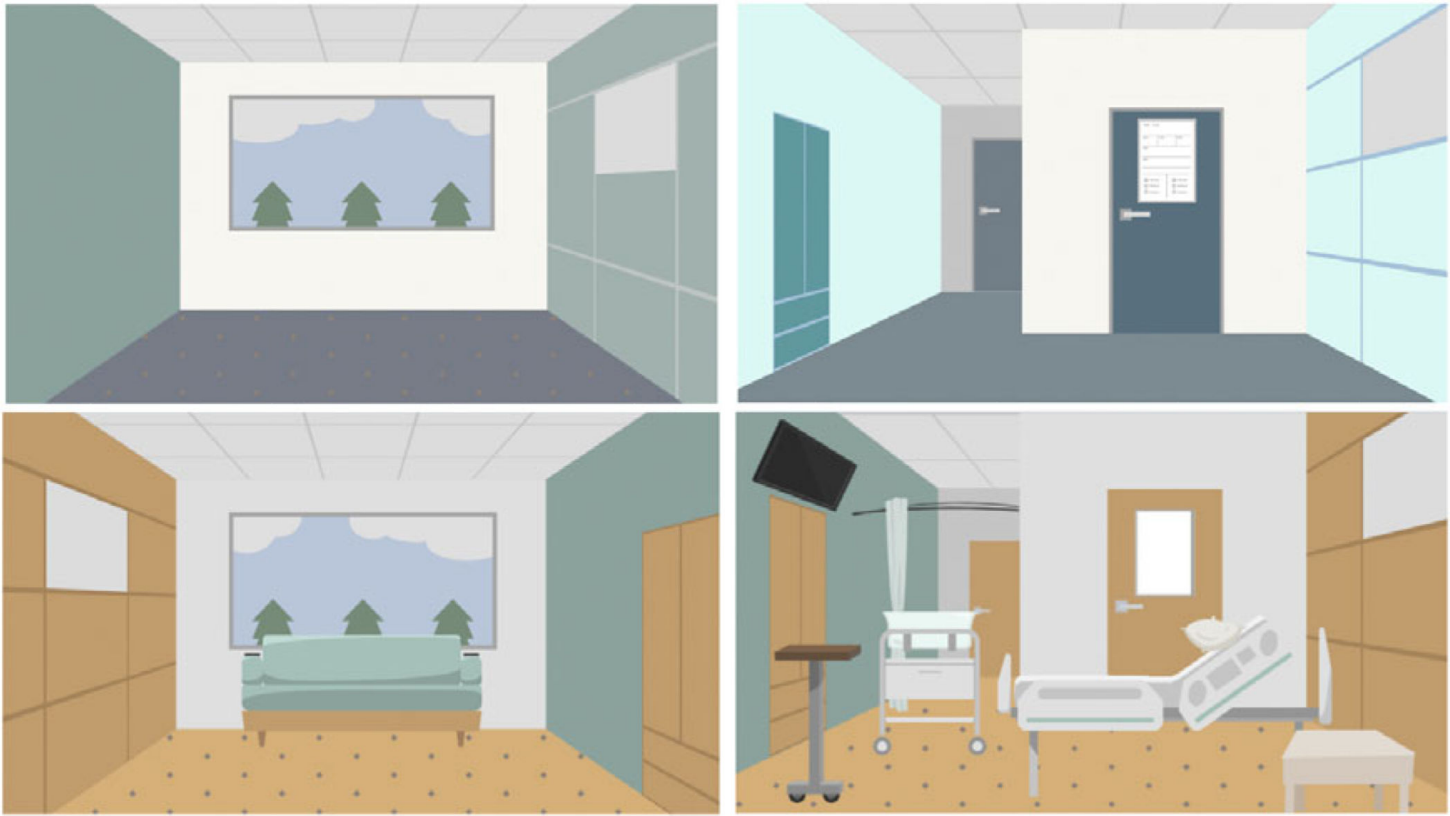
Exploration of various environmental styles and color palettes with different levels of detail.

**Figure 3.**
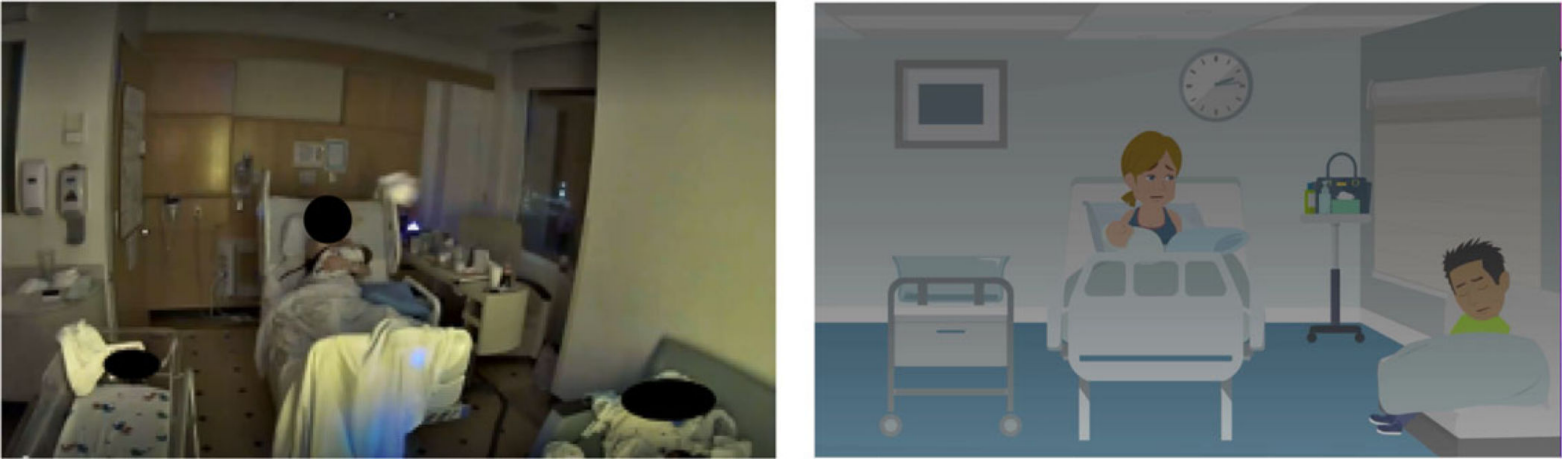
Left image (deidentified screen shot from a video file). Right image (minimalistic visualization of the video for the digital story).

The visual style of the digital stories was influenced by the observed environment and patient-participants. The initial plan was to comprehensively depict the environment by including various real-world assets and elements from the research observations (Figure 2). However, a simpler and more minimalistic approach was ultimately chosen to focus the viewer’s attention on the characters and their interactions (Figure 3). This approach aimed to avoid distracting the audience with irrelevant information, enabling them to focus on the digital stories’ primary objective of conveying events, interactions, emotions and the message of the story. Therefore, the digital stories were designed by incorporating only the key assets and environmental elements that could influence the storyline (i.e., hospital furniture and equipment). It was also important for the characters to reflect the race and ethnicity of study participants, including skin tones and body shapes. Simultaneously, it was important to protect participant confidentiality and exclude any potentially identifiable information.

#### Technical considerations..

The development of digital stories is reliant on appropriate tools and technologies. High-quality digital stories can be time-consuming and require software licenses, which can pose constraints, particularly when designers have limited experience in animation. The design researchers leading the digital stories had basic familiarity with the required techniques involved creating digital stories. Thus, two primary considerations were taken into account when selecting software for the project: the ease of use and the possibility of collaboration of the design researchers and potential future members who would be engaged in developing the digital stories. Several potential software options were compared before selecting Vyond, an online animation software (Vyond 2023). This software allowed for collaborative work and had a short learning curve, avoiding the need to learn complex editing features. The incorporation of features such as a library of visual assets and flexible character settings facilitated efficient production of digital stories, and also ensured scalability of the project in the long run. These features enabled transfer of information to future research partners or members that were interested in assisting with the design and development of the digital stories.

**Figure 4.**
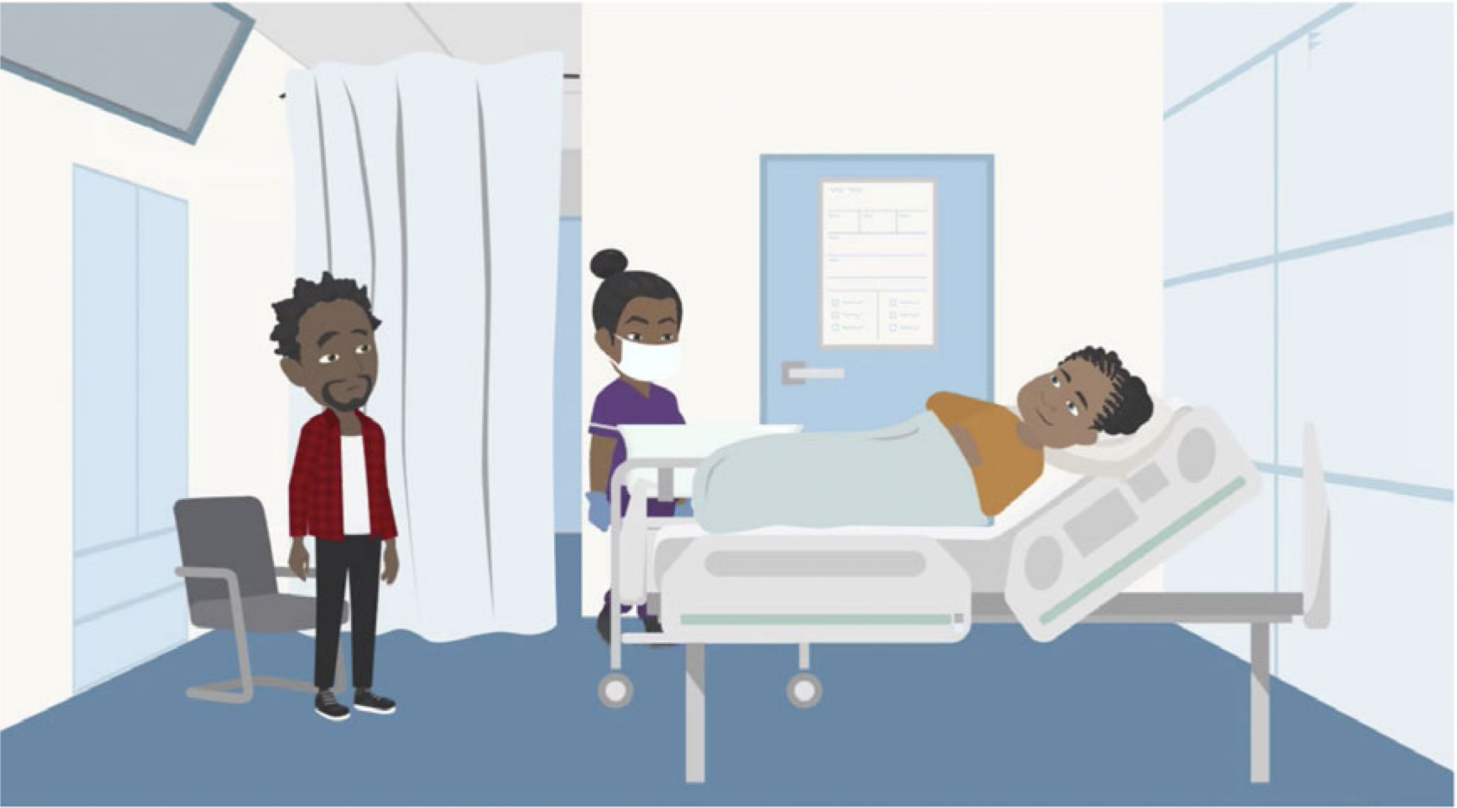
Ensuring visual styles of characters matched the ethnic, cultural and physical features of participants.

Vyond provided a variety of built-in visual styles, such as preset actions and expressions, and flexible settings for character design, such as skin tone, hair and body shape. These settings were beneficial in assigning the ethnic, cultural and physical features of the participants (Figure 4). Moreover, this platform facilitated the creation of a library of visual assets that reduced subsequent production time significantly because of shared common assets. Although Vyond was determined to be a suitable software for the development of digital stories, it was not without limitations. One limitation was the inability to create realistic characters. Additionally, the software had limited facial expressions and movements available in the character design library. These limitations required the team to employ creative solutions, explore alternative approaches to replicate certain emotional experiences, and it precluded animation of close-up, nuanced character positioning and movements.

Regarding animation voice similarities with study participants, research partners who shared the same race and ethnicity as the characters contributed to the voice-over recordings. These IRB-approved team members reviewed the original data before recording the scripts, paying attention to the emotional context, tone and feeling of the conversations.

#### RtD phase 3: evaluate

The evaluation phase of this study involves two primary processes: iterative feedback from the research partners and a participatory feedback session with the majority of the involved members of the research team including design researchers, partners and other subject matter experts.

#### Iterative feedback from the research partners..

In this study, the design researchers collaborated with research partners who were experts on maternity care. Regular weekly meetings were held to iteratively evaluate and revise the digital stories in terms of priority events, storyline, storyboard, visual style, scope, message, use cases and voice-overs. This collaborative process was crucial in refining the digital stories and maintaining validity, which were intended to serve as standalone artifacts for generating discussion.

Thorough documentation was a critical component in employing the RtD approach for developing the digital stories. This involved recording feedback, challenges and discussions, as well as documenting the steps taken to develop the digital stories. The rigorous documentation process ensured that all essential information was captured and could be accessed throughout the project’s duration. This promoted transparency and accountability in the development process and also facilitated the generation of knowledge regarding best practices for developing digital stories in the healthcare context.

#### Participatory feedback session with research partners..

A participatory workshop was conducted on September 23, 2022 with a group of 18 research partners and graduate students from diverse backgrounds and expertise. The session included two activities to assess and collect feedback on the video itself and to generate ideas for use cases or applications of the digital stories. The session was facilitated by the design researchers.

#### Activity 1: video review..

The first activity focused on feedback about the content, language and visual style of the animated stories developed to date. To this end, four digital stories were presented, each illustrated a different healthcare topic: maternal pain, lactation, companion support and baby’s care. Subsequently, the individuals were provided with an open-ended questionnaire that elicited written reflections on the videos and on the potential application of these digital stories in health care (Table 3). The questions also prompted research partners to share their opinions on the strengths and weaknesses of the digital stories in terms of their format, including visual and auditory elements. Each person completed the questionnaire individually and then were encouraged to share their impressions with the larger group. Throughout the discussion, facilitators were responsible for documenting the feedback and insights shared.

#### Activity 2: alternative scenarios and role playing..

The second activity was designed to draw upon the diverse expertise of the research partners to develop alternative scenarios that could effectively address the gaps illustrated in the video stories. The activity was also designed to encourage the individuals to generate ideas about potential applications of digital stories in healthcare education. To this end, a role-playing exercise was implemented. The group was divided into four subgroups, each was assigned one of the digital stories. Two key questions were presented to guide their scenario development: “What was happening here?” and “How might the system of care be improved?”

### 2.2. Analysis

Responses were analyzed inductively in accordance with guidelines of qualitative content analysis (Creswell & Poth 2016). The responses of team members and notes taken by facilitators during both activities were transcribed into a digital table. The information was coded and grouped together to identify emerging themes and patterns. This process of analysis allowed for a more in-depth examination of the feedback and insights shared during the session and helped to identify key areas of focus for future development. All discussions were documented by the facilitators.

## 3. Results

### 3.1. Result of RtD phase 3: evaluation

#### Activity 1 results

The feedback gathered on the review of the video was grouped into two categories: Usage of digital stories for training and education and reactions and recommendations on the content and format.

#### Usage of digital stories for training and education.

The research partners who participated in the workshop strongly agreed that digital stories have the potential to be effective tools for educational purposes, such as developing training modules and promoting discussions on respectful interactions, care coordination and quality of care. Some of the comments gathered:

- Digital stories can demonstrate the importance of protecting and promoting birthing parents’ autonomy and for the companions to be included in the education and interactions.
- Digital stories can inform policies such as staffing levels and other systemic issues. One individual remarked:
  “This is great! One thing it makes me consider is whether this video (digital story) and ones like it could be developed with a focus on healthcare administration and leadership. I think sometimes challenges are primarily considered based on how providers or care teams can improve, but I think this kind of tool could be valuable for advocating for adequate staffing.”
- Digital stories could empower healthcare professionals and students by building empathy and enabling them to comprehend and reflect on the perspective, emotions and experiences of patients and their families, including instances when healthcare professionals are not present in the room. For instance, one individual stated that
  “This (digital story) is a great way of empowering healthcare team members and students to think about what might be going on when they leave the room. I think this would be great for students across healthcare.”
- The use of digital stories is a valuable approach that has the potential to enhance healthcare education and foster greater empathy among healthcare professionals by highlighting the gaps and emphasizing areas for improvement.
- Digital stories could be made available through online open-access platforms or incorporated into existing interprofessional curriculums to maximize their reach and impact. One individual shared,
  “We all want to provide good care but sometimes don’t realize how we are acting until someone brings it up, or we have it in a video recording (digital story).”
- The use of digital stories can extend beyond their current scope to shed light on how patients’ and healthcare team members’ experiences, implicit biases and backgrounds can influence the quality of care throughout the hospital system. For example, one participant mentioned,
  “These videos would encourage providers to reflect on their own treatment of patients’ pain and how race and other factors (ex: substance use history) might influence that treatment.” Another individual stated, “Digital stories highlight the discrepancies with Black parents and the small (but big) encounters and injustices the families face. This should be shown widely.”
- Digital stories have the potential to be utilized in patient education and facilitation guides, aiding the discharge process and providing information on available resources.

#### Reactions and recommendations on the content and format..

Throughout the workshop, the research partners identified strengths in content and format. They appreciated the structure where the introduction provided context for the experiences of the characters in the stories, as well as the use of summary takeaways, reflections and questions to encourage discussion. They noted that the digital stories effectively demonstrated intersecting and compounding issues, and that the verbatim wording used was powerful.

Other positive attributes that were recognized included the voice, noting that they effectively conveyed realistic feelings and emotions. They praised the use of visual details, such as pain visualization, clock showing the time, facial expressions and gestures, which complemented the narrative and supported the main message of the story. The combination of the voice-overs with the visual elements of the digital stories helped to create a more immersive and engaging experience for the audience.

In terms of areas for improvement, one issue raised was the need to reinforce parts of the stories by providing further descriptions of technical terms, such as bilirubin or specifics about medical procedures. Additionally, it was suggested that providing closure and bullet points at the end of the videos to show opportunities for improvement based on each scenario could be a useful approach. Other suggestions included focusing on the healthcare system competency, showing comparisons of experiences, examples of effective and ineffective interactions or potential solutions to some of the challenges identified in the stories.

#### Activity 2 results

The second activity identified potential use cases of these stories in health care. The feedback gathered during this activity was grouped into three categories: envisioning ideal/desired future, addressing upstream contributors and creating questions and prompts for discussion.

#### Envisioning ideal/desired future.

One of the groups developed an alternative scenario to an interaction shared through a digital story. The group envisioned the desired future or ideal interactions that could have prevented the challenges and negative consequences presented and developed a role-play script. This role-playing was an enjoyable experience and modeled more positive patient experiences. The activity prompted discussion about the challenges faced by healthcare professionals in providing quality care. The group members shared their experiences and provided creative approaches to address the existing gaps in the system.

#### Addressing upstream contributors.

Two of the groups focused on considering potential upstream contributors of the incidents presented in the digital stories. For example, one of the groups mentioned that patients feeling that their voices are consistently not heard or being taken seriously by healthcare professionals can be the underlying reason for some of these experiences. In response, some team members suggested that asking patients about their priorities with open-ended questions can be a valuable approach to facilitating equitable and quality care. This type of interaction can demonstrate that patients’ voices and needs are heard and valued.

The group also discussed additional topics to improve communication between patients and healthcare team members, such as the physical positioning of clinicians in relation to patients and family members. One individual stressed the significance of healthcare professionals sitting while communicating with patients. This small gesture can significantly impact the patients’ perceptions of power dynamics by being at the same eye level and being focused with attention. It was suggested that incidents presented in the digital stories could potentially be prevented through more transparency with information on topics such as pain management, both prenatally and postnatally. Adopting a more proactive approach may contribute to more timely and meaningful shared decision-making. Steps to make information more accessible and comprehensible instead of relying on assumptions may minimize the likelihood of conflicts or misunderstandings, especially with sensitive topics like opiate medication use.

#### Questions and prompts for discussion.

One of the groups created their own set of questions that would facilitate developing alternative scenarios for these stories, such as “what is the problem in the digital story,” “why is it important for health,” “what can the healthcare team do,” and “how might the individuals depicted cope.” They suggested that the videos can also include links or QR codes to documents for best practices. Another area that was discussed by this group was the concept of patient-family encouragement from their healthcare team members, as part of active support.

### 3.2. Results summary

Based on documentation gathered during the design and findings of the evaluation phases, a summary of the stages for developing a digital story to share healthcare experiences was created. Table 4 provides a chronological summary and description of these stages.

## 4. Reflection and discussion

The collaborative RtD production process was a human-centered approach for sharing patient experiences through digital storytelling. During the RtD process, feedback from research partners and content experts, as well as rigorous documentation of the steps, challenges and opportunities, were used to create a conceptual framework for the animations. This framework was developed to guide the development of digital stories in healthcare settings, with the goal of provoking discussion and generating knowledge while preserving patients’ and other research participants’ privacy. The framework was designed and tested iteratively based on knowledge generated from developing 12 digital stories from naturalistic videos (Postnatal Patient Safety Learning Lab 2023). It outlines the steps required for creating each digital story, from writing a goal statement to finalizing the videos with audio. The framework includes the research team’s perspective on the minimum number of iterations required for each step, for developing a meaningful digital story. Also, supporting information and considerations for each step highlight critical aspects of each stage.

The development of Table 4 was based on a starting point of having access to IRB-approved study video and audio data. However, in healthcare settings, original data are often collected using other methods, as the sole source or through mixed-methods. Therefore, the expanded framework includes considerations for the development of digital stories using various sources of data, including interviews, shadowing, observation and workshops or focus groups. Table 5 presents inputs and activities through the stages of digital story development.

Although Table 5 is presented here in a stepwise approach, the collaborative process is iterative. Additionally, flexibility and the ability to adjust the framework components according to the specific objectives are crucial. Depending on the project’s goals and implementation plan, the digital stories can be utilized as standalone artifacts or incorporated into a multi-level intervention. Although these 2D digital stories were created using currently available technologies, it is important to consider that the healthcare industry has shown an increasing interest in the use of emerging technologies such as virtual reality, augmented reality and mixed reality (Saedi and Rice 2020; Saedi, Einifar, & Barati 2021; Panda et al. 2021). This opens up the potential for creative approaches to immersive digital storytelling that can move beyond didactic education toward immersive and active engagement.

### 4.1. Strengths and limitations

Although the digital stories and the proposed/conceptual framework were tailored for the maternity healthcare and maternity care context, the insights are transferable to other settings involving multifaceted and sensitive health-related subject matter. Furthermore, the framework developed through the RtD approach employed in this study offers practical guidance to researchers and practitioners on how to create compelling digital stories. The resulting deliverables are promising for prompting discussions, training purposes and enhancing patient-focused healthcare systems.

Our experience developing digital stories were based on recorded observations, which represent only one type of healthcare data. As with all observational research, participant behavior may have been influenced by their participation. However, the Hawthorne effect was likely minimal over the extended period of time and because of the participants. Moreover, the video and audio data were collected from one hospital in the southeast of the United States during the COVID-19 pandemic, which means the examples may not be generalizable to all birthing facilities or contexts. Additionally, the prioritization of digital stories was led by the research partners. The issues most salient and modifiable may differ by audience. Partnering with more clinicians, patient representatives and community-based health equity leaders would strengthen the process and deliverables.

### 4.2. Future work

The feedback received from research partners involved in this study indicates that digital storytelling can be a powerful tool for generating discussion around systems of health care. Sharing patients and families’ lived experiences through animating, with emotions and their words is a promising approach for building more shared understanding (Mojtahedzadeh et al. 2021). There is also an opportunity to elevate the stories of healthcare professionals, so the structural contributors to their practices and the emotions they feel, including moral injury, can be shared. Moreover, comparing digital stories with narratives featuring real-life characters to observe how emotional responses are elicited and how problems are framed differently could be a valuable area for future research. Although the considerations and requirements for creating stories with real-life characters may differ, this exploration can open up a promising avenue for comparison and exploration of various forms of storytelling within the context of sensitive health data.

Future research can evaluate the impact of digital stories in interprofessional education and clinical quality improvement. Additionally, stakeholder feedback on the digital stories would inform the extent to which the current animations are culturally relevant and accurately depict the experiences of patients and families. Assessing how research participants feel that their experiences can be leveraged to improve systems of care is important. Research results like the digital stories we have created offer nuanced insight into patient-family and healthcare team interactions, which can inform both immediate healthcare practice change and system reform. Additionally, by testing the proposed framework with various tools and platforms for digital storytelling and using diverse data sources such as interviews, focus groups and workshops, the knowledge gathered through this study can be transferred to a broader range of healthcare-related areas.

## 5. Conclusion

This study articulated the use of HCD and digital storytelling to share the experiences of patients and their families in the postnatal unit. This article provides an overview of the rationale, approach and impact of digital storytelling for provoking discussion and creative problem solving among healthcare professionals in the context of maternity care. This work employed an RtD method. We started with observational data and used a design approach to translate observational data into animated stories. This intentional approach had the potential to meaningfully engage stakeholders, to increase empathy and inspire action.

Furthermore, a key strength of this study was the interdisciplinary team composed of designers, clinicians and researchers. The partnership allowed for the development of new research approaches and the application of their respective skills, tools and methods for transdisciplinary collaboration. Through the human-centered perspective, the team generated new knowledge on the use of creative media and artifacts, such as digital stories, to convert sensitive and rich health-related data into prompts for ideation and problem solving.

The research through design study outlines a human-centered approach for digital storytelling to promote discussions, education and enhance healthcare systems, thereby fostering respect, equity and support for maternal care.

## Figures and Tables

**Table 1.** Five steps of RtD

RtD Step	Description

Select	Teams choose a research problem or design opportunity worth exploring (wicked or messy problems, having multiple agendas driven by different stakeholders) and decide on a new material, context, target population, societal issue or theoretical framework to focus on through an iterative process.
Design	Using insights gained during Select to develop a solution or prototype through testing and refining with end-users or stakeholders. This can include conducting a literature review, fieldwork, workshops and exploring ideas to understand the problem framing and offer a new perspective. The team should document their design moves, critique their initial framing, and evaluate and refine their ideas iteratively.
Evaluate	Assessing the artifact based on the selected RtD practice and research question. The team should reflect on what they learned and disseminate the research through publications, videos or demonstrations.
Reflect and disseminate	Reflecting on the design process and outcomes, and sharing findings through academic publications, presentations or workshops to advance knowledge and inform future design projects.
Repeat	The final step is to repeat the process to optimize results.

**Table 2.** Digital story prompts

Prompt	Descriptor/supporting questions

Researcher partner name	This information was obtained to enable follow up discussions with the team members or research partners that filled out the form.
Video participant ID(s)	Deidentified information and data file name
Timestamp(s) of event(s)	Time of event(s) (video number)
Goal of the animation?	Describe the audience (healthcare professionals, leaders, etc.) Who is this animation for and why?
What is the main message of the animated story?	What should the viewer glean from the animation? What should the animation communicate?
Character descriptions	Describing the key characters in the story (birthing parent, companion, midwife, nurse, etc.) Who are they? What are their roles?
What is the set up?	What background information does the viewer need to know?
What is the conflict?	What is the problem that the characters are experiencing?
How does the story end?	Is there a resolution? Is there a take-away? Is there an unanswered question? Is there a call-to-action?
Narrative description of scene(s)	Share some details about what happens in your chosen story. (Significant positive or negative interactions)
Note important actions, quotes or sounds that are most descriptive or stand out	Highlight the key discussions, interactions, events or environmental factors. These elements should be critical to the storyline.

**Table 3.** Prompts used in the participatory feedback session

Prompts for activity 1: What are your thoughts on existing animated videos?

How can these videos be utilized in healthcare education? (When/How/For whom?)
What are your thoughts on the strengths and weaknesses of the presented animated videos, considering both the format (look and feel) and content?
**Prompts for activity 2: Assist us in developing new animations or exploring alternative scenarios.**
Review the future potential scenarios
Write down your reflections on each scenario
Share your suggested scenarios for future animations
Select the scenarios you can help with.

**Table 4.** Summary and description of stages for developing a digital story

Development stages	Description

**Create a goal statement** (minimum of two iterations)	Define the goal for developing the digital stories, audience, audiences needs and use cases of the stories. How will these stories be used? Share the goal with the team.
**Create a template** (minimum of two iterations)	Design a template to allow team members to identify and record key events from the source based on the defined goal. The template should be easy to work with and include details such as summary of the event, insights, main characters, message, environment and setting.
**Create repository of key events** (minimum of two iterations)	Code the data based on the goal and categorize the information for easier future access. This stage can highlight the reference images, videos, key quotes, core behaviors, attitudes or issues.
**Identify the events**	Select the events worth sharing and evaluate potential takeaways. The event should convey a specific message to your audience.
**Write the script** (minimum of two iterations)	Write the script based on the original event and repository of key events (e.g., video clip, notes, quotes, etc.). Identify the key moments of the event to include in the script. The script is not purely a transcription of the dialogues or quotes. Also identify appropriate references for further transferability.
**Storyboard** (minimum of two iterations)	Create a storyboard based on the key ideas of the script. Determine the main visual assets such as characters, furniture, spaces and primary items in the environment. Prioritize essential assets to the story. The age, ethnicity, race and cultural concordance of the characters should be considered.
**Low fidelity animation** (minimum of two iterations)	Create the animation based on the script to test the scenario and refine the storyline including the look and feel. Use the audio prototype and get a sense of timing and overall flow. Are the details accurate? Does the feel and tone align with the original event and intent? Is the context appropriate?
**High fidelity animation** (minimum of two iterations)	Refine the visual details. The focus here is on timing and transitions so the pieces come together.
**Audio**	Add the voice-overs and ambient noises. The audio should be recorded for each character considering racial, ethnic and language concordance.
**Final video**	Contextualize it with other videos or prepare it as an independent video.

**Table 5.** Expanded framework for digital storytelling using various sources of data

		Source format		

Development stages	Naturalistic videos	Interviews	Shadowing and observations	Workshop and focus groups

Create a goal statement	Include additional details related to the primary reason, process and strategies for gathering the naturalistic videos	Include additional details related to the interview setting and objective and role of participants as stakeholders (patient, healthcare provider, expert, etc.)	Include additional details related to the observation setting, key participants and the primary objective of the observation/shadowing.	Include additional details related to the goal of the workshop/focus group and the role of participants as stakeholders (patient, healthcare professional, expert, etc.)
Create a template (vignette) to record key events	Include additional details related to naturalistic videos such as time frame, background narrative, key artifacts in the scenario, participants mood or feelings.	Include additional details related to the reference interview, the interviewee’s background and the potential option for member checking with clarifying questions.	Include additional details related to observation time, images and notes from the observation and contact information of observers for clarifying further details.	Include additional details related to involved members in the focus group or the workshop, potential mood board or visual references, feel of the scenario, option for member checking with clarifying questions.
Create repository of key events	Include visual references from videos, video clips, nonverbal cues (gestures, facial expressions, body language or postures). Information related to cultural or ethnic concordance, mannerism.	Mood boards created based on the scenario by the team and research partners (can include quotes, descriptions of emotions, environments or characteristics of people). Information related to cultural or ethnic concordance, mannerism.	Potential images from the setting or artifacts in the environment. Information related to cultural or ethnic concordance, mannerism.	Mood board created based on the scenario by the team and research partners (can include quotes, descriptions of emotions, environments or characteristics of people).
Identify the event	Minimum level of Medium level of interpretation for evaluating potential takeaways.	Medium level of interpretation for evaluating potential takeaways.	High level of interpretation for evaluating potential takeaways (highly open to interpretation).	Medium level of interpretation for evaluating potential takeaways.
Write the script	Member-check with the research partners, team members, video participants (if possible).	Member-check with the research partners, team members and interviewee.	Member-check with the research partners, team members, participants (if possible).	Member-check with the research partners, team members and workshop/focus group participants (if possible).
Storyboard	Refer to the naturalistic videos.	Refer to the mood board.	Refer to potential images or notes from observation.	Referring to the mood board.
Low fidelity animation	Review by design, healthcare professionals and research partners and study participants (if possible).	Review by design, healthcare professionals, research partners and interview participants (if possible).	Review by design, healthcare professionals, research partners and study participants (if possible).	Review by design, healthcare professionals, experts’ team and selected members of workshop/focus group participants (if possible).
High fidelity animation	Review by design, healthcare professionals and research partners and study participants (if possible).	Review by design, healthcare professionals, research partners and interview participants (if possible).	Review by design, healthcare professionals, research partners and study participants (if possible).	Review by design, healthcare professionals, experts’ team and selected members of workshop/focus group participants (if possible).
Audio	Refer to the naturalistic videos. The inclusion of cultural, ethnic and ethical considerations should be based on the project’s objectives.	Refer to the mood board. The inclusion of cultural, ethnic and ethical considerations should be based on the project’s objectives.	Refer to potential images or notes from observation. The inclusion of cultural, ethnic and ethical considerations should be based on the project’s objectives.	Refer to the mood board. The inclusion of cultural, ethnic and ethical considerations should be based on the project’s objectives.
Final video	Contextualize it with other videos or prepare it as an independent video.	Contextualize it with other videos or prepare it as an independent video.	Contextualize it with other videos or prepare it as an independent video.	Contextualize it with other videos or prepare it as an independent video.
